# TRIPS to Where? A Narrative Review of the Empirical Literature on Intellectual Property Licensing Models to Promote Global Diffusion of Essential Medicines

**DOI:** 10.3390/pharmaceutics14010048

**Published:** 2021-12-27

**Authors:** Shiri Mermelstein, Hilde Stevens

**Affiliations:** Institute for Interdisciplinary Innovation in Healthcare (I3h), Université Libre de Bruxelles (ULB), 1050 Bruxelles, Belgium; shiri.mermelstein@ulb.be

**Keywords:** access to medicines, drug costs, LMICs, innovation, patent pools, voluntary and compulsory licensing, trade-related aspects of intellectual property rights (TRIPS)

## Abstract

Governed through the World Trade Organization Agreement on Trade-Related Aspects of Intellectual Property Rights (TRIPS) since 1995, the current medical R&D system requires significant trade-offs between innovation and high monopoly prices for patented drugs that restrict patient access to medicines. Since its implementation, few amendments have been made to the original TRIPS agreement to allow low- and middle-income countries (LMICs) to facilitate access by generic manufacturers through flexible provisions, such as compulsory licensing and parallel import. Although a useful policy tool in theory, the routine use of TRIPS flexibilities in LMICs in the procurement of new essential medicines (EMs) is regarded as a ‘last resort’ due to strong political response in high-income countries (HICs) and new trade agreements’ restrictions. In this context, access-oriented biomedical Public-Private Partnerships (PPPs) have emerged. More recently, leading multilateral health organizations have recommended different types of intellectual property (IP) interventions, voluntary biomedical patent pools, as strategies to reduce prices and increase the diffusion of novel EMs in LMICs. Nevertheless, the recent Ebola and COVID-19 outbreaks highlight growing concerns regarding the use of TRIPS flexibilities and the limited success of voluntary mechanisms in promoting access to medicines in the Global South amidst health crises. This review aims at describing the state-of-the-art empirical research on IP-related options and voluntary mechanisms applied by emerging PPPs to guarantee timely and affordable access to EM in LMICs and reflect on both models as access paradigms. Some suggestions are put forward for future research paths on the basis of these analyses and in response to contemporary debates on waiving key IP rights on COVID-19 therapies, diagnostics, and vaccines.

## 1. Introduction

Since 1995 the biomedical R&D system has been increasingly globalized due to the establishment of the World Trade Organization (WTO) agreement on Trade-Related Aspects of Intellectual Property Rights (TRIPS), which requires nations to provide minimum standard intellectual property (IP) protection for new technologies, including pharmaceutical products. IP provisions restrict the use and marketing of new drugs and grant exclusive rights to patent holders for a period of 20 years to help offset the high costs required to research and development (R&D) and incentivize investments in future clinical innovation with high uncertainty. That said, throughout the biomedical innovation cycle, IP rights may also hinder the possibility of inventor collaborations, innovation activities, production, and optimal global diffusion of affordable life-saving drugs [1]. Prior to the TRIPS agreement, patenting practices for pharmaceutical products varied widely and were less common in low- and middle-income countries (LMICs) that relied on access to generic drugs manufactured in emerging markets [2]. Shortly after TRIPS was introduced, concerns were raised by public stakeholders that the agreement would result in dramatic price increases of patented essential medicines (EMs) [3] that will disproportionally affect individuals and governments in the Global South [4,5]. The 46 least developed countries (LCDs) according to the United Nations classification were exempted from granting or enforcing patent protection on medicines until 2033 (LDCs Waiver). In 2005, few amendments were made to the original TRIPS agreement to allow all other LMICs with little pharmaceutical manufacturing capacity to facilitate access to medicines by generic manufacturers through flexible provisions, namely compulsory licensing of medicines and government use of patents. TRIPS allows member states to temporarily bypass patent holder’s protections and issue compulsory licenses (CLs) assigned to government use or local private company to produce a patented product or compound for the purposes of non-commercial domestic use or to export it to a developing country that lacks manufacturing capacity (Article 31*bis*). It can be granted on any ground, among which public health emergencies, stockouts, unaffordable prices, and patent holder’s refusal to license [6].

### 1.1. Barriers to the Effective Use of TRIPS Flexibilities 

CLs are considered the key TRIPS-related flexibility for national governments in LMICs and played a central role in bringing down the price of patented medicines to treat HIV/AIDS and, more recently, Hepatitis C virus (HCV) infection, by allowing mass production of generic substitutes (Box 1) [7]. Nevertheless, the use of CLs for domestic production in LMICs is increasingly restricted and regarded as a measure of last resort due to a firm political opposition from high-income countries (HICs) and new stronger IP frameworks known as “TRIPS-plus” provisions in new multinational free trade agreements such as the recent Trans-Pacific Partnership Agreement [7]. Prior to the global pandemic, attempts by LMICs to issue CLs for domestic use were met with trade pressures, such as being included in the US Trade Representative’s annual watch list which monitors nations “unfairly issue, threaten to issue, or encourage others to issue compulsory licenses” and notes that CLs should only be used in “extremely limited circumstances” [8]. Importantly, unlike CLs for domestic production, CLs for s according to Article 31*bis* have only been used once, when a Canadian manufacturer exported generic versions of patented HIV/AIDS medicines to Rwanda in 2008 [9]. While effective in theory for countries lacking manufacturing capacity, Article 31*bis* was deemed largely non-operational, and many countries have not yet incorporated this option into their national legislation [10]. 

Of note, patents are only one aspect of IP in the global biomedical R&D. During the 1990s, access-to-medicines advocates often considered patents as the main problem to solve, whereas the “know-how” and trade secrets are often the main barriers to be considered today, as they may further delay generics’ entry to the market [11]. These include clinical trial data and regulatory exclusivities, and all other forms of non-patent exclusivities [11].

This brings us to the core problem preoccupying governments, health organizations, and companies: as pointed out by numerous prominent authors and health organizations, the current biomedical R&D system as governed through TRIPS requires significant trade-offs between incentives to innovation (IP and market exclusivities) and the high prices that restrict patient access to medicines [1,12,13,14]. Medicines entering the market are a result of cumulative innovation and a collaborative effort between a network of public and private entities. The majority of new medicines result from publicly subsidized early-stage academic research, often additionally financed through governmental innovation grant programs such as the Horizon Europe or the US National Institutes of Health (NIH) [15,16]. However, final high-cost development and commercialization steps are often funded by pharmaceutical firms themselves after they buy the IP rights from research institutes.

Box 1 **The impact of IP regimes and use of TRIPS flexibilities on access to medicines.**

A wide range of studies have looked at the complex impact of implementing TRIPS and newer stringent free trade agreements regimes and the use of TRIPS flexibilities on access to medicines from the perspectives of economics, public health, and law. Key findings from recent studies and three systematic reviews [17,18,19] are outlined below and detailed in Table S1.

*Trade Treaties and Access to Medicines*: Islam and colleagues [17] have recently reviewed 16 studies that evaluated the size of the effect (ex-post studies) or predicted the likely impact (ex-ante studies) of IP frameworks on access to medicines. Negative effects of changes to IP policies such as the implementation of TRIPS or stronger IP framework requirements on various indicators of access to medicines such as product launch, price, treatment uptake, and overall health expenditure. The authors concluded that stronger IP regimes, namely frameworks that include extensions of clinical data exclusivities, tend to block competition in the pharmaceutical market and thus incur high societal costs caused by monopoly prices for patented products. These findings indicate that newer bilateral IP agreements might pose a threat to the legal space that TRIPS intended to provide to allow earliest possible generic entry by extending data exclusivity and requiring each generic manufacturer to produce its own clinical data for the application to regulatory authorities before market authorization. In addition, a systematic review of the literature on patent expiry and prices by Vondeling and colleagues [18] found that across high-income-countries (HICs), drug prices substantially declined (by about 44% to 90% of originator price) in the few years after patent expiry and generic companies’ entry. A newer analysis by the same researchers tracked 250 patented drugs in the Netherlands and found that the median drug price dropped by 41% 4 years after patent expiration [20].

*TRIPS Flexibilities as Safeguards to Public Health*: The use of CLs has *peaked* in the years 2004–2008, in conjunction with the Doha declaration and increasing public moral outrage over the death toll of HIV/AIDS [7]. In total, prior to the COVID-19 pandemic, there have been at least 100 attempts of CLs and government use of patents by 35 countries [7]. Most of them focused on HIV/AIDS medicines, except a few instances. The economic and overall societal benefits of implementing TRIPS flexibilities are well-documented in the international literature. For example, in Thailand, the use of 7 generic drugs produced after CL to treat HIV/AIDS and several types of cancer saved the healthcare system approximately $370 million over 5 years [21]. Another recent systematic review, by Urias and Ramani [19], included 16 pre-post studies covering a total of 24 CL events occurring from 2003 to 2012 in eight countries in three continents, most commonly for patents on small molecule HIV/AIDS drug formulations. Most of the studies looked at generic entry and drug prices in different countries. Their striking results indicate that after approval, CL issuance events were associated with a 66.2% to 73.9% decrease in prices [22]. However, there were only 3 successful events of CLs from 2016 until the outbreak of COVID-19 pandemic in 2020, of which 2 were in HICs (for Shionogi to treat HIV/AIDS in Germany and Celgene for leprosy and tuberculosis in Russia) and only one in an upper-middle-income country (sofosbuvir to treat hepatitis C virus in Malaysia) [23].

In the current system, to return their investment in development, companies charge a monopoly price for new patented medicines, which is often much higher than the cost of production [14,24,25,26]. The method is based on the notion that other nations that were not involved in the R&D and commercialization activities (involuntary) contribute to its financing by buying patented medicines at high prices [14,15]. Nevertheless, in many high-income countries involved in R&D, such as the US and the EU, the public often pays twice for innovation: first through taxes and then through high medicine prices. Moreover, large companies often deploy so-called ever-greening strategies to maximize their profit by extending protection periods for patents about to expire through registering dubious follow-on patents on variations of the original drug (incremental innovation).

TRIPS created an incentive system that does not meet the need for investments in new health technologies that are marginally profitable such as therapies for diseases common among the poor and rare diseases, nor does it meet the challenges of accessing treatments for people and populations with limited purchasing power [25]. In the Global North, national health services such as those in the UK and the Netherlands are starting to limit entry of overpriced medicines; meanwhile, 85% of the population lives in the Global South, where prices of biomedical products are often as high but even less tuned for the population’s needs and incomes [27]. Prior to the COVID-19 pandemic, it has been estimated that about half of the global population has no regular access to EMs [28].

### 1.2. Voluntary Licensing and Patent Medicines Patent Pools as New Access Paradigms

With an out-of-balance medical IP system and the use of TRIPS flexibilities being restricted, access to EMs in LMICs is increasingly relying on donation-based biomedical Private-Public Partnerships (PPPs) between two or more multi-sectoral bodies focusing on the pooled procurement and delivery of medicines [29,30]. Similarly, access-oriented product-development partnerships and governmental research grants have emerged to facilitate early-phase R&D activities towards neglected and poverty-related diseases, areas where the launch of new drugs may be marginally profitable for private companies [12,25,30]. Among the non-for-profit PPPs, there are GAVI, the Vaccine Alliance, Drugs for Neglected Diseases Initiative (DNDi), and The Global Fund to Fight AIDS, Tuberculosis, and Malaria, all funded mainly by the UK, USA, and Bill and Melinda Gates Foundation [20]. Meanwhile, in face of continued public pressure and controversies, prominent pharmaceutical companies have adopted voluntary licensing (VL) of IP rights under pro-access terms as a key strategy to facilitate generic entry in low-income countries (LICs), mainly for HIV, HCV, and from 2021, COVID-19 health products. However, Access to Medicines Index has recently estimated that less than half of existing patented EMs are covered by pharma companies’ access strategies in poorer countries, and the majority of late-stage R&D projects for new EMs are not supported by future access plans [31,32].

**Figure 1:**
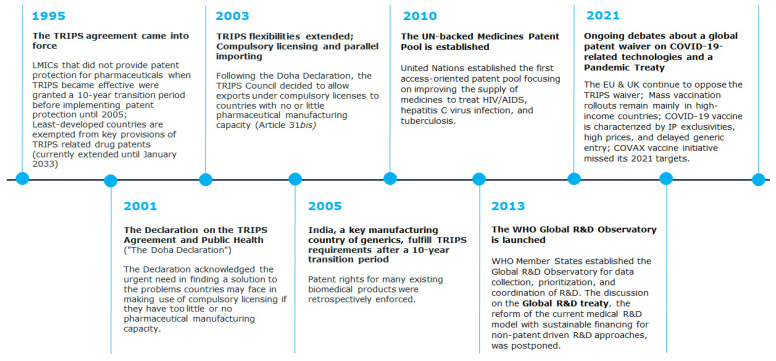
Timeline of global agreements and initiatives related to IP and access to medicines.

In this context, leading bodies such as the World Health Organization (WHO) and the Lancet Commission on Essential Medicines Policies have recently recommended on biomedical patent pools as a different type of voluntary practice to promote early generic entry and overcome IP barriers to health [1]. In 2010, the Medicines Patent Pool (MPP) was established by UNITAID as the first centralized VL platform for EMs in LICs (Figure 1). The MPP negotiates with patent-holding pharmaceutical companies for public-health-driven licenses agreements and then grants royalty-bearing sub-licenses to qualified generic manufacturers (sub-licensees) in developing countries that supply active pharmaceutical ingredients (APIs) and end products to certain LICs at prices closer to the marginal production cost. In addition to its direct contribution to facilitating faster launch of generic products in LMICs, the MPP also publishes the full texts of its licenses and constructed the largest database on the status of EMs patents in LMICs (*MedsPal*). Both databases are a precedent of transparency in the pharmaceutical field and potentially contribute to more informed decisions by governments and international donors regarding the procurement of legally produced generics at lower costs [33,34].

The MPP initially focused on patents related to HIV and expanded its model to license products related to HCV, tuberculosis (TB) medicines in recent years. Between 2010 and 2021, the MPP has signed agreements with ten patent-holders and more than 25 generic manufacturers for 13 priority HIV antiretrovirals (ARVs), one HIV technology platform, three HCV direct-acting antivirals (DAAs), and one TB treatment. Over the next five years, the MPP strategic plan is to become a general patent pool for patented small molecules on the WHO Model List of EMs [35]. In addition, as we discuss later, since May 2021, the MPP expanded its mandate into the licensing and knowledge transfer of COVID-19 antivirals and vaccines [35].

Currently, the MPP licenses with branded firms usually cover about 90–100 countries, including all sub-Saharan African countries and Djibouti [36]. Inclusion in the MPP territory mainly depends on countries’ income group, previous licensing status, and negotiations with originator firms, regardless of country-specific medical needs or demand shocks [36,37]. Hence, despite their high prevalence of HIV or HCV, several middle-income countries (MICs) such as Thailand and Malaysia are excluded from MPP licenses and price discounts and must continue to rely on TRIPS flexibilities to access low-priced generics [38,39]. South Africa is an exception, and although it is a MIC, it is often included in voluntary licenses (VLs) and non-enforcement agreements for antiretroviral drugs on the basis of its high HIV prevalence.

From an economic perspective, few authors noted that the MPP is substantially different from patent pools in other fields, such as software and electronics [40]. In these fields, patent-holders are often interested in joining a for-profit patent pool because they need licenses for the patented technology of other patent-holders to develop their own products. In the biomedical fields, the MPP and previous similar initiatives were established by the beneficiaries of the technology, such as civil health organizations patient groups, to promote access to specific products in specific geographical regions [40]. Originators might be interested in licensing through the MPP platform since it can support the originator firms in expanding their market in LMICs by lowering the costs of preparing and enforcing new IP licensing contracts with multiple generic manufacturers in different regions. For generic manufacturers, the MPP offers a waiver of royalties from sales of any new pediatric formulations included in the pool and often offers royalties at reduced rates (Table 1) [34]. 

### 1.3. Rationale and Aims

During the discussions on the COVID-19 vaccine patent waiver proposal put forward by India and South Africa, the persistent debate about the current design of the global medical R&D system and its adverse consequences on public health and economic inequalities has peaked [46]. As of December 2021, TRIPS waiver proposals on the COVID-19 vaccine met strong opposition from the European Union (EU), and only two events of CLs for COVID-19-related therapies were recorded in HICs [42]. Both the Ebola and COVID-19 pandemics highlight the concerns regarding the implementation and use of TRIPS flexibilities in LMICs and the limited success of donations and VLs in increasing the diffusion of health technologies in the Global South amidst a global emergency [47]. Advocates of the MPP claim that pooled licenses are particularly effective for promoting access to EMs in LMICs [48]. Critics argue that these tools might be misused by pharmaceutical companies for virtue signaling and rent- seeking purposes, undermining the use of TRIPS flexibilities and additional policy safeguards related to IP [49]. In times of the largest global health crisis in more than a century and in light of the ongoing policy debate, it is crucial to assess the effectiveness of IP countermeasures currently available for policymakers, civil organizations, and companies.

While there is a large body of evaluative literature on the effects of the use of TRIPS flexibilities on the diffusion of new drugs in LMICs, the theoretical and empirical literature on biomedical patent pools and additional voluntary IP mechanisms is new and limited in scope. The broad aim of this review is to explore the state-of-the-art of the empirical support for the use of medicines patent pools and bilateral voluntary IP agreements to promote timely and affordable access to EMs in LMICs. Subsequently, we attempt to identify remaining gaps and make some suggestions regarding future research.

## 2. Review Scope and Methods

We conducted a non-structured narrative review of the empirical evidence addressing voluntary IP licensing models to increase affordability and availability of new and existing EMs. Although systematic reviews are placed higher on the hierarchy of evidence-based public health in answering specific research questions, non-systematic reviews play an important role in addressing a topic in wider ways [50,51]. The rationale for opting for a narrative review in this study is to outline what has been previously published on the topic and seek new study areas not yet explored [50,51]. Nonetheless, each step of the review was informed by the Preferred Reporting Items for Systematic reviews and Meta-Analyses (PRISMA) reporting guidelines [52,53].

The literature databases PubMed (MEDLINE), Scopus, and EconLit were searched in October and December 2021 to identify relevant publications in English, without limits of date of publication or geographical settings. Additional gray literature and scholarly materials, including working papers, dissertations, and book sections, were manually searched using institutional websites, Google Scholar, and snowballing through review of references in the identified publications. Full information on MeSH terms, keywords, and Boolean operators used and search strategy developed for each database is provided in the Supplementary Materials (Table S1) to improve reproducibility of results [52]. All publications that reported results from an original quantitative or qualitative research study on the impact of voluntary IP models on indicators of access to medicines were included. We excluded expert opinions, patients’ and advocacy groups’ views, and policy analyses articles since we were only interested in empirical evidence.

After removing duplicates using Covidence and Mendeley online platforms, SM conducted title and abstract screening. In the next stage, both authors independently participated in full-text review and selection and reached a consensus on the studies to be included.

Data on research objectives, methodology used, data sources and type (proprietary or publicly available data), population, period and medicines covered, outcomes of interest, controls, main findings, and key limitations were extracted from selected references using an Excel form. Findings from the included studies were synthesized using tables and a narrative summary.

## 3. Results

### 3.1. Study Selection and Characteristics

**Figure 2:**
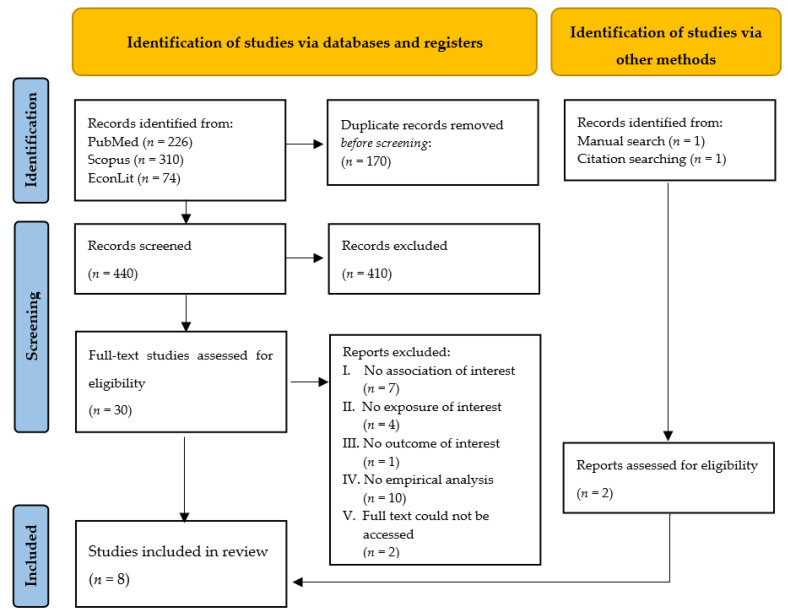
PRISMA 2020 flow diagram of the article selection process.

The reviewed literature is situated at the intersection of several broader strands of the literature in economics of innovation, patent pools, and healthcare economics, namely healthcare affordability. A PRISMA flow-diagram [53] reports the selection of papers for inclusion and exclusion at the different stages of the review process (Figure 2). The structured and manual search strategy identified 612 titles to review (Figure 2). A total of 170 duplicates were removed using Covidence, and 440 abstracts were screened. In total, 32 articles have been considered eligible for full-text analysis. Applying the selection criteria, 24 articles were excluded (Table S2).

We identified eight papers for review; all were published between 2017 and 2021 and used data ranging from 2004 to 2020 (six years prior to the establishment of the MPP in 2010 and a decade after). All studies performed quantitative analysis, including four studies that applied a quasi-experimental approach using difference-in-difference [34,36,54,55] two ex-ante impact assessment models [48,56], one study that focused on database construction and descriptive analysis [6], and one study that estimated the effectiveness of current voluntary measures in achieving the SDG goals of HCV elimination by 2030 [57]. Two studies investigated both licenses administered via the MPP alongside licenses agreed bilaterally between major pharmaceutical companies and manufacturers in developing countries [6,55]. Despite similarities in their overall design, the studies differ in their methodologies, choice of medicines and outcome variables, scope of LMICs included in the analyses (between 35 and 129 countries), data sources used, and methods used to assess access outcomes. About half of the studies looked at HIV/AIDS antiretrovirals included in the pool [6,34,36,48] due to data availability. Others considered medicines for HCV [55], specific case studies of medicines for HIV and HCV [56], or a broad combination of EMs for HIV, HCV, and TB [54]. Most studies reported on impact on the total number of licenses signed through the pool, drug quantities purchased by LMICs and donors, and shares of generics [6,34,36,54], three studies estimated past and projected cost savings [36,48,56], and only two studies looked on treatment uptake [55] and potential public health gains [56]. One study also investigated the effects of VL on cumulative innovation [36], and another included exploratory analysis of the effects on information asymmetries [34]. Key study characteristics and results are summarized in Table 2 and Table 3.

### 3.2. Pooled and Bilateral VLs and Access to Medicines

In the following section, we describe key findings, organized according to the following themes: (1) generic drug diffusion, including the share of generics and total quantity of drugs purchased; (2) actual and projected cost savings; (3) public health impact; and (4) follow-on innovation.

#### 3.2.1. Generic Drug Diffusion

In a recent National Bureau of Economic Research (NBER) working paper, Galasso and Schankerman [54] asked whether patent pools promote global drug diffusion, that is, how quickly and how many generic products become commercially available in different countries. To this purpose, they used rich MPP data on HIV, HCV, and TB medicines that the organization aimed to license when the pool was formed in 2010 to construct a treatment group (medicines covered by the pool) and a control group (medicines for which bargaining with the pool started but failed). Using a difference-in-difference analysis, the authors found that inclusion in the MPP increases the likelihood of patent licensing deals for the related products and that when compared to non-MPP product-country pairs, the probability of having at least one license to a generic firm increases more than five-fold. Nevertheless, the effects were heterogeneous: they were larger for small, non-sub-Saharan countries and smaller in LMICs with high prevalence of HIV, possibly because bilateral deals between pharmaceutical companies and governments are more likely there regardless of the pool [54]. Their second result is that increased licensing through the MPP is only weakly correlated with actual drug launch in countries covered by the agreement, implying that MPP may only be interested in launching their generic product in a subset of countries included in the pool according to their market potential.

##### Generic Shares

Interestingly, a cross-sectional study by Beall and Attaran [6] that linked data on patents, procurement and use of legal IP flexibilities by developing countries found that of the access-oriented mechanisms considered, VLs (either bilateral or administrated by the MPP) appeared to be applicable to the largest volumes of ARVs generic procurements where patent protection had been estimated (over 78%), compared with LCD waivers (21.7%), CLs (0.25%), patent non-assert policies (0.37%) and other non-specified mechanisms (21%). Accordingly, they conclude that VLs may be major facilitators of generic access where patents have been granted. Nevertheless, the authors note that while CLs were used sparingly in their sample, they may still be the best mechanisms among the flexibilities for some countries and medicines [6]. Two quasi-experimental studies looked at the change in the share of generic versus originator purchases of a specific HIV drug [36] or HCV drug [34] in a specific country-year following its inclusion MPP. Both studies findings indicate that the post-period increases in the total number of units sold by generic companies over the total number of units sold for an MPP drug are clear and substantial (7–20%) given the already high generic coverage in developing countries during the sample period [34,36]. In a more exploratory analysis, Martinelli and colleagues [34] suggested that MPP databases further contribute to increased generic entry by eliminating asymmetric information on the IP rights status of drugs across geographical markets.

##### Total Quantity and Price of Generic Drugs Purchased 

Results of three quasi-experimental studies indicate that after inclusion in the pool, the total quantity of EMs purchased by procurement agencies (government or health organizations) in MPP territories is likely to increase a few years after its inclusion compared with LMICs that were not included in the agreement [34,36,54]. In all studies, data on sales is deemed insufficiently rich for detailed analysis of drug price development for following their inclusion in the pool. Given that inclusion in the MPP leads to a substantially higher volume of products sold, yet its effect on total market revenue is minimal, Galasso and Schankerman [54] interpreted that MPP inclusion may be associated with a decline in drug market prices (computed as sales/volume). Wang’s [36] price development analysis suggests that shortly after the VLs for several HIV drugs were reached by the pool, drug price per patient-year fell by about US$87 (pre-post price ratio is not provided), mainly from a substantial reduction in generic prices.

#### 3.2.2. Actual and Projected Cost Savings

Wang [36] retrospective cost-benefit model also found that the inclusion of HIV/AIDS medicines in the MPP significantly increased welfare compared to no-MPP cases between 2010–2017. Her estimations of consumer and producer surplus increase by US$0.7–1.4 billion (8.6–18.9%) and US$181 million (4.5%), respectively, far exceeding the MPP operating cost of US$33 million in the same period. Although not comparable, two impact assessment models used by MPP projected the societal cost savings from pooled licensing through the pool due to improved entry of generic manufacturers. Focusing on HIV medicines, the earliest assessment by Juneja and colleagues [48] from 2017 estimated that actual cumulative savings generated by MPP licenses between 2010 until 2028 would reach US$2.3 billion, while the pool’s operational costs are expected to reach only US$50 million over the same period. Morin and colleagues [56] have recently updated the MPP assessment model to account for additional assumptions on the effect of drug prices on procurement decisions in the absence of pooled licensing when drug prices are usually higher. Their model predicted that between 2017 and 2032, over US$3 billion saved through the MPP license for dolutegravir-based HIV treatments compared with the counterfactual scenario, and US$107 million saved for daclatasvir-based HCV treatments between 2015 and 2026. For comparison, the authors note that cost savings for dolutegravir are equivalent to the total HIV investments made by The Global Fund in Nigeria, South Africa, and Zimbabwe since 2002 [56].

A different method to understand the effect of voluntary participation of pharmaceutical firms in licensing agreements is provided in Assefa and colleagues’ [57] capacity-to-pay study in seven African countries covered under a bilateral VL and tiered pricing agreement with Gilead for sofosbuvir-based DAA [40]. Their findings suggest that under the present agreement with Gilead, to provide universal access to these therapies, countries will have to exceed their annual health budget by additional expenditure ranging from 4% (South Africa) to about 400% in Cameroon.

#### 3.2.3. Public Health Impact

Only one ex-post study we are aware of by Simmons and colleagues [55] estimated the actual impact of VLs for several DAAs, either initiated via the MPP or agreed bilaterally between a prominent pharmaceutical company and several generic manufacturers, on annual treatment uptake in a large number of LMICs included in the agreements. By applying a quasi-experimental analysis on detailed epidemiological data, the authors were able to show that VLs were associated with an increase in treatment uptake of about 54 per 1000 individuals diagnosed with HCV. This effect was realized a few years after inclusion in the pool and increased over time. Although not comparable, Morin and colleagues [56] predicted that the inclusion of daclatasvir-based DAAs in the MPP will yield an additional uptake of 428,244 (range 127,584–636,270) patients, 4070 (range 225–6323) deaths averted between 2015 and 2026. In the case of dolutegravir-based HIV treatments, their model predicted an additional uptake of about 15 million patient-years between 2017 and 2032, and 151,839 (range 34,575–312,973) deaths averted with the MPP license compared with the counterfactual scenario; these projections correspond to the total of AIDS-related deaths in 2019 in five populous African nations with a high burden of disease (Nigeria, Mozambique, Uganda, Kenya, and the Democratic Republic of the Congo) [56].

#### 3.2.4. Follow-On Innovation

Following past literature on the relationship between IP policies and cumulative innovation (e.g., [37]), Wang [36] also demonstrated the potential positive spillover effects of the MPP on follow-on innovation. These findings suggest that licensing through the MPP slightly increases Phase III clinical trials for new indications or better bundling of MPP compounds as well as new drug approvals (branded and generic products) between 2005–2018. Follow-on trials were initiated by firms inside or outside the pool; branded firms inside the pool reallocated investment to test new compounds that can further complement existing drugs included in the pool, whereas firms outside the pool conducted more late-stage trials for new drug cocktails with pooled compounds. Overall, sub-licensors obtained more generic drug approvals with pool-associated compounds, especially for sales in developing countries. One possible explanation for these effects is that when branded firms sign a deal with the MPP, it signals their “openness to IP diffusion” [36] (p. 10), thus lowering the litigation risks to research institutes.

## 4. Discussion

### 4.1. Summary of Main Findings

The present review highlights that there has been a notable increase in the number of studies that have explored the multifaceted effects of collaborative IP models, particularly through the MPP, on health and economic outcomes in LMICs. This research attention might reflect how IP and high prices of medicines increasingly affect individuals and societies. A decade after the establishment of the MPP, new findings, mostly tested by using quasi-experimental economic models and health impact assessment simulations using the MPP publicly available data, suggest that patent pooling fostered generic manufacturing, leading to increased generic market shares, total quantity of essential HIV and HCV medicines purchased in countries included in the agreements and improved treatment uptake volumes. The strength of these associations, however, is mostly determined by country characteristics, drug type, time intervals, and health and market outcomes considered. As discussed above, the effects of VLs on actual prices and treatment uptake across countries are challenging to capture. Yet taken together, there is considerable evidence that VLs, namely through the MPP, lower prices through generic competition and improve uptake of EMs. These findings were overall consistent with the broad literature on the role of generics in improving access to medicines [18,19,20].

Moving one step forward, Wang [36] suggested that pooled licensing may also act as an incentive for further R&D investments. These findings relate to a stream of research in innovation policy attempting to quantify the size and direction of the effects of stronger global IP policies on clinical research activities and cumulative innovation [58]—the process of inventors building on the efforts of many earlier inventors—in developed [59,60] and developing countries [61]. Wang’s [36] findings indicate that more collaborative IP frameworks through patent pools, rather than more exclusive frameworks, potentially boost innovation. Relatedly, an exploratory analysis by Martinelli and colleagues [34] attempted at providing evidence that the MPP’s commitment to making transperent data on medicines patent status across LMICs available to the public may balance information asymmetries in the traditionally opaque pharmaceutical market [62] by reducing uncertainties about IPRs (clarifying a potential freedom-to-operate situation) and thus encouraging the procurement of generics in LMICs.

### 4.2. Challenges in Measuring Voluntary Licensing Impact on Access to Health

Reviewed studies repeatedly reported certain methodological challenges. First, data offer only a few years of observations since most VLs have only been agreed upon during the past decade, and they are mainly addressing medicines for HIV/AIDS and only very few other diseases. A more general problem in the field is the scarcity of transparent data on prices and volumes of generic or branded medicines procured, as well as epidemiological data in some LMICs. Some authors conducted field work to collect price data from multiple public authorities [36,57], while others used proprietary data [54]. In both cases, data were insufficient for a more nuanced analyses on prices and quantities, including the exploration of channels through which the observed effect occurred. Lack of transparent price data also impedes cross-national comparisons. Additionally, possible chain effects of VLs such as the impact of MPP licensed generic products on prices for branded equivalents have not been captured yet. Therefore, Morin and colleagues [56] argued that the overall effect of VL on access might be underestimated.

Conversely, the evaluations of VL rely on several assumptions while some important contextual factors may have similar effects on drug diffusion, price, and uptake, and therefore might overestimate the effects of VL on access to innovation. Some hard-to-model factors, such as comprehensive transmission dynamics and context of VL negotiations, may have affected quantity purchased, treatment uptake, and price regardless of the VL agreement [56]. Factors such as the patent grant dates and the due diligence of a patent portfolio should be included in all analyses. As argued by Martinelli and colleagues [34], patent holders’ willingness to sign a VL agreement might be influenced by the patent expiry date, as older patents are associated with less profits and lower interest of firms to enforce their patents. Previous evidence from studies on IP and access to medicines show that new medicines are more likely to be affected by data exclusivity, new patents, and patent linkage, whereas older medicines might be more influenced by patent term extension [63].

### 4.3. Future Research

In the following, we outline several open questions that need to be addressed in future studies to create a more complete picture of the role of voluntary IP models in improving EM accessibility. In particular, as briefly mentioned above, access-oriented biomedical patent pools differ in many ways from patent pools in other technological fields, and more empirical and theoretical research is required to generate a systematic evidence base for the concept from the perspective of public health, global health governance, and economics of innovation.

#### 4.3.1. Measuring the Effects of Different VL Practices in Different Settings

To gain a deeper understanding of the conditions under which such licensing might be most beneficial, future studies need to examine different VL models in varied geographical settings and explicitly test potential adverse effects of VL. First, given the distinct characteristics of voluntary agreements negotiated through the MPP and those granted through bilateral agreements outside the pool between two for-profit companies, and the variation in licensing practices (non-exclusive versus exclusive), duration and geographical scope, it is necessary to compare and separately assess the effects of these practices on access-related outcomes.

Secondly, more studies in specific regions and domains of diseases are needed. Although the general potential of the MPP in facilitating generic production and uptake has been documented across a bundle of LICs included in the VL agreements, study designs often mask unique characteristics of countries and regions. One exception is the recent study by Galasso and Schankerman [54] that investigated the effects across different characteristics of included studies. This could motivate future studies to offer nuanced comparisons and case studies of specific areas.

Subsequently, as with any evaluation of any intervention, it is vital to consider and test potential negative results related to each VL approach, alongside all the potential benefits. Several studies reported weak effects of VLs, but more attention is needed to directly test and report potential negative effects. For example, Martinelli and colleagues [34] describe findings from patent pools in other fields, raising concern that patent pooling could foster monopolistic power of originators by limiting the number of generic manufacturers included in the license, blocking further innovation, or by collaboration among competitors in the pool to maintain high prices [62,64,65]. We do not know, for example, what are the potential effects of the pool’s agreements on the affordability of patented EMs in many MICs excluded from the pool despite high burden of disease. These countries are obliged to grant patent protection for pharmaceuticals and are more vulnerable to political pressure when they attempt to invoke TRIPS flexibilities, and they are often excluded from receiving donor support based on income criteria [1]. Some critics, such as the Third World Network [49,66], additionally argue that VLs can be used by pharmaceutical companies for rent-seeking purposes and abusive marketing practices, covering for weak patent portfolios that would have been otherwise opposed by many countries, thus allowing the free procurement of generic companies. As developed countries’ national governments have not yet played a central role in regulating VLs [67], more empirical research is needed in these areas to inform policymakers.

#### 4.3.2. Qualitative Studies

Another critical issue requiring future research attention is the lack of qualitative studies evaluating the role of biomedical patent pools and negotiating practices from the perspective of policymakers, health providers, and generic manufacturers in LMICs. Future research is needed to provide insights on how the MPP (and similar initiatives) negotiate specific access-oriented IP clauses and characterize the knowledge transfer models and how these frameworks are applied in practice. Similar studies were previously conducted in the field of early-stage research private-public partnerships [15,30,68]. Detailed qualitative information could inform the expansion of the MPP model and the creation of similar pools to include more priority health technologies, qualified generic manufacturers and additional countries based on their level of unmet medical need rather than income alone.

#### 4.3.3. Affordability and Budget Impact

Except for Assefa and colleagues’ [57] study, studies to date were mainly directed on the scope of savings through patent pooling and bilateral VLs, yet affordability is only implied. Despite their high value, financial, and public health gains, even generic EMs might not be affordable in some countries, at the collective or individual level, resulting in shortages or financial hardships. At the collective level, biomedical product affordability for the public sector or health organizations depends on the price of the product (defined by supply, demand, and market structure), the available budget, and the fiscal space [1]. Although there is no consensus on the definition and methods to assess affordability or financial hardships [1,69], it is an issue for future research to investigate affordability and governmental budget outcomes using measuring tools developed by the WHO and others.

#### 4.3.4. Collaborative IP, TRIPS Flexibilities and Health Governance during COVID-19

The introduction of a wide range of innovation policies and the scale of globalized networks of public-private collaborators, supported with unprecedented public funding, contributed to the accelerated development of COVID-19 diagnostics, vaccines and therapies [70,71,72]. Nevertheless, the challenges and motivation to ensure a more equal health technology diffusion have never been clearer [47]. As of December 2021, less than 6% of people in LICs are fully vaccinated, despite donations from wealthy nations where more than 66% of the population received at least two vaccine doses [73]. Without reaching global herd immunity, new variants are emerging and could send the world back to square one.

In October 2020, India and South Africa, with the support of many developing nations, requested a temporary waiver of key TRIPS provisions for several patented COVID-19 health technologies to ensure rapid generic production and availability in LMICs. Opponents of the waiver, including the EU, UK and Canada, and pharmaceutical industry representatives, argue that such changes to IP regulations would be counterproductive for technology sharing and have a serious chilling effect on inventors [74]. They maintain that existing access-oriented IP measures, including country-by-country TRIPS flexibilities and collaborative strategies based on advanced market commitments (including differential pricing, pooled licensing, and patent non-assertion declarations) are sufficient to protect public health interest and promote access to COVID-19 health innovation worldwide. However, according to hundreds of international health organizations, researchers and the majority of WTO Member States, this is hardly the case [47,74].

At the outset, we note that to date there have been no CLs or pooled licensing agreements through the MPP for any vaccine [42]. Prompt implementation of TRIPS flexibilities and VLs is likely to foster the low-cost production of generic versions of small molecule drugs in LMICs, such as the overpriced antiviral therapeutic Remdesivir, due to patent protection being a key barrier in their creation. Nevertheless, while the EU often portrayed CLs as the main IP countermeasure available for limited-resource governments during public health emergencies, in practice, only two affluent countries, Russia and Israel, were granted CLs for COVID-19 related therapies according to Medicines Law & Policy’s TRIPS Flexibilities Database [42]. In this context, the MPP has recently expanded its mandate into licensing new COVID-19 antiviral drugs developed by two major pharmaceutical companies [75,76].

To increase production and global diffusion of high-quality generic versions of COVID-19 vaccines, a higher level of collaboration and multiple additional non-patent IP and trade secrets need to be addressed through technology transfer, on top of IP licenses [77]. In response to the soaring inequities in the production, procurement, and distribution of the COVD-19 vaccines, mainly in Africa, the MPP in collaboration with the WHO and African international partners have set out to establish the mRNA Vaccine Technology Transfer Hub in South Africa in July 2021 to ensure sustainable, local vaccine production by 2024 [78].

Little empirical evidence has been generated so far on IP flexibilities and population health during the pandemic. More studies are needed to map and better understand the scale of use, effectiveness, and limitations of various collaborative IP models, industry-led responses, and policy tools to overcome market exclusivity barriers to affordable COVID-19 biomedical technologies across LMICs amidst the pandemic.

For example, the (relatively) rapid introduction of emergency innovation policies across countries, the expansion of the MPP mandate, and industry-led mechanisms in response to the unmet medical needs across a wide range of countries resulted in countless unplanned natural experiments [79]. Future quasi-experimental studies (similar to those reviewed above) could exploit the variation in IP strategies to examine whether or not current IP interventions have a sufficient mitigation impact on access to small molecule medicines (without having a chilling effect on follow-on innovation). Notably, some data can only be collected now by agencies across the world [79]. Such analyses will require detailed longitudinal data from multiple public sources (e.g., MPP open-access licensing database, patent opposition and TRIPS flexibilities) and less transparent data from countries and consortiums. Indeed, rigorous research using quasi-experimental techniques cannot eliminate all uncertainties arising from changes to IP policies [79]. Still, it has the potential to clarify some of the causal relationships between less exclusive innovation strategies and health technology diffusion, affordability, or follow-on innovation needed for evidence-based design of new IP policies and pandemic preparedness strategies.

The topic of IP interventions to increase COVID-19 vaccine diffusion ignites further conceptual and methodological questions for researchers. So far, 22 vaccine developers have directed their efforts into increasing production capacity in their own facilities or through sub-licensing arrangements with at least 130 manufacturers based in 45 countries [80]. Of these, 76/96 arrangements were agreed between vaccine developers and manufacturers based in HICs [80]. However, lack of transparent information on the scale and degree of patent-holder restrictions on markets eligible for distribution under these arrangements [80,81] is likely to hold back future research. In addition, civil organizations have identified 100’s other manufacturing facilities in 35 developing countries that could potentially be used to manufacture COVID-19 vaccines if a TRIPS waiver was implemented [82]. It might be useful to (i) model the potential effect of TRIPS waiver on vaccine production, and (ii) assess current IP interventions in comparison with those previously applied to increase the generic production of other patented vaccines considered essential as per the WHO list [3]. One interesting case could be GAVI’s work on reducing the high costs of the human papillomavirus (HPV) vaccine, which can prevent several common cancers and virtually eliminate cervical cancer, which currently claims the lives of more than 300,000 women each year; approximately 90% of those deaths occur in LMICs [82]. Although the HPV vaccine was first introduced in HICs in 2006, as of 2020, just 13% of girls aged 9–14 globally were vaccinated against HPV and around 80 LMICs are yet to introduce the vaccine [82].

Lastly, most COVID-19 health technologies, such as MPP-licensed molnupiravir, were invented in US and UK universities, supported with taxpayer money [15,72,83]. Preliminary findings suggest minimal access-related measures were set in place by public institutes to promote fast and equitable access in LMICs [15]. More studies are needed to assess the extent of inclusion of access conditions (open innovation, knowledge transfer policies, end-product affordability, and the availability of clinical trial data) in COVID-19 related research grants and consortium contracts throughout the phases of clinical R&D.

### 4.4. Review Limitations

The present review excluded articles written in languages other than English and so important findings and perspectives may have been missed. In addition, the review aimed at presenting a narrative synthesis of recent findings and, importantly, did not attempt at computing effect sizes or include a risk of bias assessment for included papers given the heterogeneity of study designs and objectives. Several other closely related issues could not be addressed in this scope. These include more attention to the complexity of knowledge transfer processes and availability of prequalified manufacturers in LICs, the role of national IP and competition law, and the assessment of other IP-related interventions occurring at pre-approval stages. For instance, non-commercial R&D initiatives with IP commons targeting missing EMs, such as the Global Health Innovative Technology (GHIT) governmental initiative in Japan and product development partnerships such as DNDi, which already developed six new treatments for neglected diseases [1]. These models prioritize open innovation and condition public investment in early stages of development on access for the poorest [25], ensuring that products are developed on a “no gain, no loss” basis [84]. Moreover, these initiatives also shed light on the costs of biomedical R&D, including the cost of failure, suggesting it might be much lower than the high figures reported by pharmaceutical companies to justify the high cost of medicines [25].

## 5. Concluding Remarks and Policy Implications

In response to the increased interest in interventions to balance IP, our objectives for this paper were two-fold: (i) to review the available evidence for the effectiveness of voluntary licensing practices, mainly through patent pooling, and (ii) to identify knowledge gaps and future research directions.

In the current global medical R&D system, governed mainly through the TRIPS agreement, generic drugs often enter the market following the expiration of lengthy patents and market exclusivities. The use of TRIPS flexibilities in LMICs to facilitate affordable and faster access to EMs is increasingly restricted. To compensate for the deficiencies in global policy, non-commercial R&D and pooled IP initiatives have emerged, largely funded by a few high-income countries and the Bill and Melinda Gates Foundation. The present review identified a number of recent studies that tracked access to generic EMs in LMICs before and after the imposition of VLs through the MPP or outside the pool and evaluated these interventions from a public health and economic perspective. Overall, these studies indicate that in 11 years since the first biomedical patent pool establishment, it fostered generic diffusion of several EMs for HIV and, more recently, HCV, benefiting millions of people and achieving impressive financial savings for developing countries and international health organizations. One clear implication is that biomedical patent pools should be encouraged by governments.

We highlighted some areas for future exploration to define success formulas and pitfalls of alternative IP models, such as (i) measuring the effects of different VL approaches in different settings, (ii) conducting qualitative interview studies with key stakeholders, (iii) assessing the effects of patent pools on biomedical products affordability at the collective level, (iv) mapping and examining the effectiveness of collaborative IP models and use of TRIPS flexibilities during the COVID-19 pandemic and in relation to global health governance.

Lastly, for the avoidance of misinterpretations of findings, we reiterate that while the option of voluntary participation of prominent pharmaceutical in licensing agreements through one operating patent pool is beneficial, it cannot provide a sustainable and comprehensive solution to the underlying fundamental problems and policy failure of a health innovation system relying on market exclusivities. No single IP option can [6]. The MPP is highly effective in some circumstances and for some LMICs, but it is unlikely to generate a critical mass of patents outside the pool’s (and public awareness’) areas such as rare diseases, mental health, and most non-communicable diseases [40]. These issues were discussed extensively elsewhere over the last two decades [1,5,25,40]. Important policy implications are that the present review’s findings and the growing support for biomedical patent pools should not undermine the need to protect the widest possible range of policy options to overcome IP barriers at the multilateral levels, including full use of all TRIPS flexibilities and policy countermeasures such as the application of strict patentability criteria and patent oppositions. Ideally, with the expansion of the MPP to other EMs, a patent holder’s refusal to license an EMs to the MPP should satisfy the condition for granting a compulsory license under TRIPS Article 31, which requires the grantee to have made efforts to obtain authorization from the patent-holder on reasonable commercial terms and conditions [7].

The public debate on the topic of tackling IP barriers to promote health equity is being stifled by the assertion that “there is no alternative” to the current medical R&D system based on market exclusivity—a key cause to problems preoccupying most governments. One main concern is that, in the times before COVID-19, policymakers did not heed the robust empirical findings in the IP-health literature or misinterpreted them. These concerns are crucial to global health today amidst the ongoing pandemic, which has already intensified the existing disparities in living standards and health outcomes in many parts of the world [83]. Echoing the Lancet Commission’s proposal [1], we restate the need to construct a new global policy framework to incentivize and reward innovation of new EMs through countries’ contributions, in a tiered method proportionate to their economies, to enable the development of missing EMs and de-linkage of large investments associated with R&D and the price of newly developed medicines.

## Figures and Tables

**Table 1:** Key TRIPS flexibilities and voluntary IP practices to promote generics entry of essential medicines in LMICs.

Mechanism	Description	Example	Publicly Available Data
LDCs waiver	LCDs are exempted from key TRIPS provisions related to medical technologies, which may allow them to purchase or produce generics even for patented drugs	Bangladesh’s large pharmaceutical industry accounts for around 1% of gross domestic product and supplies almost the entire domestic market and export to >100 countries. Around a fifth of generic drugs produced in the country under the TRIPS waiver are patented in other countries [41]. There is a concern that when Bangladesh will leave the UN LDC category in 2024, and no longer be eligible for the waiver, it will hinder the country’s technological development and a substantial rise in health costs [41].	TRIPS Flexibilities Database (Medicines Law & Policy) [42]
Compulsory licenses (CLs) and government use of patents	Under TRIPS, a country is authorized to license IP rights for a patented medical product for domestic production without the patent holder permission to increase access to a particular drug	Malaysia, an upper-middle-income country, was excluded from VLs of costly patented DAAs despite a high burden of HCV infection (prevalence of 2.5% among the adult population in 2009). Patented sofosbuvir, a core of DAAs, was sold in Malaysia at about US$11,000, while its production was estimated to be below US$136 [24]. In 2017, the government issued a compulsory license, which was also followed by the country’s inclusion in bilateral VLs. In about two years, generic versions of sofosbuvir under the compulsory license brought down the public procurement price to US$300 per 12-week treatment [24,38].	TRIPS Flexibilities Database (Medicines Law & Policy) [42]
Patent opposition	National legal procedure where a third party may object to an application for registration of a trivial patent as part of an ever-greening strategy	Successful patent oppositions are common in countries with strong national legislative mechanisms for the use of TRIPS flexibilities, such as India and Brazil. A patient-led group led to the rejection of GlaxoSmithKline’s patent application in India in 2006 on the HIV fixed-dose-combination zidovudine/lamivudine, on the grounds that it was not an ‘inventing step’, but rather a combination of two existing drugs widely used in practice [43].	Patent Opposition Database (Médecins Sans Frontières) [44]
Voluntary licenses (VLs)	Bilateral, non-exclusive contractual agreements between patent-holding firms (licensors) and each generic manufacturer (licensees) which allow the supply of lower-cost generic medicines to certain LMICs in exchange for a royalty fee	In 2014–2015, Gilead Sciences licensed patents for its DAAs compounds used to treat HCV (sofosbuvir, ledipasvir, and a newer compound, velpatasvir) through bilateral agreements with 11 generic manufacturers for use in 101 countries, predominantly LMICs [1].	MedsPal (MPP) [23]
Voluntary licensing through a patent pool	The UN-backed Medicines Patent Pool (administrator) negotiates licenses for high-value EMs with patent-holding firms (licensors) to allow their production by generic manufacturers (sublicenses) in exchange for a reduced royalty fee	In 2015, the MPP signed an agreement with Bristol-Myers Squibb that allows the supply of generic versions of DAA compound daclatasvir in 112 LMICs [1].	MedsPal (MPP) [23]
Patents non-assertion declaration	In humanitarian situations and in response to access campaigns, patent holder companies may commit not to enforce patent lefts in a defined group of countries and under specific conditions, allowing a generic version to be produced	In 2009, Boehringer Ingelheim granted non-assert declarations to all generic manufacturers prequalified by the WHO in Africa and India to produce HIV/AIDS drugs containing the active ingredient nevirapine. The declaration covered 78 countries, including all African countries, low-income countries, and LDCs [45].	MedsPal (MPP) [23]

**Table 2:** Summary of Study Characteristics.

Reference	Objective	Methodology	Data Sources	Population	Period	Medicines	Main Outcomes	Controls
Simmons, Cooke, & Miraldo, 2019 [55] [Peer reviewed]	To estimate the effect of the introduction of bilateral and MPP voluntary licenses for HCV drugs on access to treatment	Difference-in-difference analysis Treatment group: countries included in the licensing for HCV treatment from either Gilead (VL) or Bristol-Myers Squibb (through the MPP) Comparison group: countries not included	Polaris Observatory—HCV epidemiology and treatment volumes, Gilead and MPP voluntary licensing agreements data	MPP-licensed LMICs (*n* = 19): 127.2 M people, average GDP per capita $8.5 K, average 9% prevalence of HCV among adult populations. Non-licensed LMICs (*n* = 16): 132.6 M people, average GDP per capita $17.3 K, average 0.9% prevalence of HCV.	2004–2016	HCV DAAs	Annual HCV treatment uptake per 1000 individuals diagnosed with HCV	Country-level fixed effects; Time-variant economic effects, health expenditure, and health system indicators; Region-specific year effects to control for unobserved time-variant factors
Wang, 2019 [36] [Preprint]	To evaluate the impact of the MPP on static and dynamic welfare: how the MPP affects generic shares in LMICs, the changes in R&D associated with the pool, and the welfare gains compared to the pool’s operating costs	Mixed-methods, including difference-in-difference analysis and cost-benefit analysis	The global fund price and quality reporting, FDA and AIDSinfo.gov	103 LMICs	2007–2017	HIV medicines	Total quantities and generic shares Changes in R&D (new trials and approval of new drugs associated with the pool) Welfare gains compared to the pool’s operating costs.	GDP per capita, Worldwide Governance indicators, HIV prevalence and age-adjusted death rates
Martinelli, Mina & Romito, 2020 [34] [Working paper]	To exploit heterogeneity in the timing of entry into the MPP across countries to estimate the effect of the pool on the market for EMs	Difference-in-difference analysis	The GPRM (Global Price Reporting Mechanism), the MPP website, and the MedsPaL (Medicines Patents and Licenses database)	The final sample included 3862 observations and 616 pairs of country/active pharmaceutical ingredient (API) included in the pool. Countries where a generic version of the MPP-API was already available and commercialized before they joined the pool.	2005–2017	HIV medicines	The annual total quantity and share of generic versions of pills of a specific API bought yearly by procurement agencies and delivered in a specific country	Fixed-country effects and a variable controls for the possibility that shifts are driven by changes in the agencies’ budgets, Ginarte Park index (a proxy for level of nations’ patent protection)
Galasso & Schankerman, 2021 [54] [Working paper]	To study how the Medicines Patent Pool affects the licensing, launch and sales of drugs in LMICs	Difference-in-difference analysis Treatment group: drug-country pairs from the MPP Comparison group: drug-country pairs—medicines that the MPP aimed to license when the pool was formed in 2010, for which bargaining with the pool started but failed.	IQVIA data on international drug products sales, MPP licensing data (including information on non-MPP products and non-MPP bilateral VLs between the upstream patentee and generic firms)	129 LMICs countries for which patent protection was in place for at least one of the sample drugs. Data on sales are only available for a subset of 32 countries, mostly middle-income countries outside Sub-Saharan Africa.	2005–2018	173 EMs for HIV, TB and HCV	Number of downstream licensing deals, launch, quantity sold and price of EMs in LMICs.	Time-varying demographic features of the sample countries (World Bank Data) Prevalence of HIV in each country per-capita health expenditure
Juneja, Gupta, Moon, & Resch, 2017 [48] [Peer-reviewed]	To estimate the savings generated by licenses negotiated by the MPP for ARVs to treat HIV/AIDS for the period 2010–2028 (the year by which patents on all of these drugs will have expired)	Cost savings attributed to the MPP were calculated by subtracting the expected price of ARVs medicines following inclusion in MPP licenses from a counterfactual situation in which the MPP does not exist. A cost-benefit ratio was calculated based on the pool’s actual and projected costs.	MPP Medspal, UNAIDS reports on patients accessing HIV/AIDS therapies, and tiered prices data by Médecins Sans Frontières	MPP impact is attributed only to countries where the MPP license had unblocked existing patent, and the country was not eligible for supply by generic producers included in any existing or planned bilateral VL	2010–2028	All 13 HIV medicines included in the pool by 2016	Projected cost savings associated with 13 MPP licenses A cost-benefit ratio	N/A
Morin et al., 2021 [56] [Peer reviewed]	To study the economic and health effect of voluntary licensing for medicines for HIV and HCV in LMICs	MPP impact assessment modeling study to examine the difference between factual and counterfactual scenarios, with and without an MPP license for two case study medicines	MPP licensees, the Polaris Observatory (market share forecasts), matched with epidemiological information from UNAIDS	All LMICs	2012–2020; 2020–2032	Dolutegr-avir (for HIV) and daclatasv-ir (for HCV)	Cost savings—drugs costs and health system costs associated with untreated disease progression. Health impact—uptake, mortality, morbidity, and adverse effects linked to HIV or HCS disease progression or the medicines used.	N/A
Beall & Attaran, 2017 [6] [Peer reviewed]	To assess to what extent LMICs that have granted patent protection on essential ARVs procure generic equivalents of those medicines, and identify which legal flexibilities (CLs, LCD waivers, VLs, and non-assert declarations) may have been most relevant for facilitating this access.	Data linkage and cross-sectional descriptive statistical analysis. The researchers cross-referenced the datasets with lists of legal flexibilities which facilitate generic access where patents have been granted.	Patent databases (USA, Canada, and international) WHO’s Global Price Reporting Mechanism ARVs procurement data in LMICs, and various institutional and academic sources tracking the use of legal IP flexibilities	85 LMICs, a total of 1924 generic procurement transactions (1.34 billion units) for a sample of ARVs	2013–2014	13 patented ARVs that were sold by a single, originator supplier in the US or Canada and are likely to be patent-protected in LMICs	Median patent coverage Alignment of generic procurement with patent protection in the exporting and/or importing country. Volume of generics purchased attributed to different legal flexibilities	N/A
Assefa et al., 2017 [57] [Peer-reviewed]	To test the hypothesis that Gilead’s bilateral VLs and tiered pricing strategies for DAAs in seven African countries will fail to achieve the SDG 2030 goal of HCV elimination and are insufficient for achieving fair and equitable access to DAAs in those countries.	A cross-sectional analysis of countries’ financial capacity to provide DAAs for HCV treatment under present VLs and tiered-pricing arrangements. Conservative estimates were used—prices for 12-weeks regimens, lowest and factory gate prices, assumed zero re-infection.	The prices used for modelling were taken from a 2016 WHO report	A convenience sample of 7 African countries with experiencing a different range of HCV disease burden and eligible for generic supply under Gilead’s VL: Egypt, Ethiopia, Nigeria, Democratic Republic of Congo, Cameroon, Rwanda and South Africa.	2016	HCV DAA’s (sofosbuvir and sofosbuvir/ledipasvir)	Financial capacity of each country to provide universal access to selected DAAs under present VLs and tiered-pricing arrangements with Gilead	N/A

**Table 3:** Summary of Key Findings: The Impact of Pooled Licensing on Access to Essential Medicines.

Design	Reference	Key Findings
**Quasi-Experimental** **Studies**	Simmons, Cooke, & Miraldo, 2019 [55]	- Voluntary licenses (through the MPP/bilateral) are associated with an increase in treatment uptake of 53.6 per 1000 diagnosed individuals in the two years after implementation (95% CI 25.8–81.5). The effects are increased over time.
	Wang, 2019 [36]	- Inclusion of country-HIV/AIDS compound pairs in the MPP increases the share of generic purchases of a compound (by about 7%). - Cost-benefit analyses show that the MPP is estimated to increase welfare substantially compared to no-MPP cases. Consumer surplus increases by $0.7–1.4 billion (8.6–18.9%), and producer surplus can also increase by up to $181 million (4.5%), far exceeding the $33 million operating cost in the same period. - Inclusion of a compound in the pool is also associated with more follow-on clinical trials, and more firms participate in the trials.
	Martinelli, Mina & Romito, 2020 [34]	- Countries under an MPP license purchased about 2.9 million more units of HIV/AIDS pills compared with countries without the license. - Inclusion of country-HIV/AIDS compound pairs in the MPP increases the share of generic purchases of a compound (by about 20%). - Exploratory analysis suggests that the MPP further increases access by eliminating asymmetric information on the IP rights status of drugs across geographical markets.
	Galasso & Schankerman, 2021 [54]	- Inclusion in the pool is associated with a five-fold increase in the probability of licensing. The effect is heterogeneous—it is much larger for small, non-sub-Saharan countries and smaller in countries with large exposure to HIV (where bilateral deals are more likely). - Inclusion in the MPP increases the likelihood of launch and total quantities sold, and reduces prices. Nevertheless, the magnitude of these effects is much smaller than the one estimated for the effect on licensing.
**Impact Assessment Models**	Juneja, Gupta, Moon, & Resch, 2017 [48]	- Actual cumulative savings from 2012 until 2015 reached USD195 million. - Between 2010 and 2028, the model predicted US$2.3 billion saved over a cost base of a little over USD 50 million over this 18-year timeframe. - A cost-benefit ratio—based on people living with HIV in any new countries which gain access to ARVs due to MPP licenses and the price differential between originator’s tiered price and generics price, within the period where that product is patented—is projected to be 1:43. i.e., for every US$1 spent on MPP operational costs, the global public health community saves US$43.
	Morin et al., 2021 [56]	- The cumulative effect attributed to MPP license is predicted to reach additional uptake of about 15 million patient-years of dolutegravir-based HIV treatments is predicted between 2017 and 2032, 151,839 (range 34,575–312,973) deaths prevented, and more than US$3 billion saved, compared with the contrafactual scenario (absence of MPP license). - For daclatasvir-based HCV treatments, the cumulative effect from 2015 to 2026 was projected to be an additional uptake of 428,244 (range 127,584–636,270) patients treated, 4070 (225–6323) deaths prevented, and around $107.5 million saved through the MPP license.
**Descriptive Study**	Beall & Attaran, 2017 [6]	- Of the public health flexibilities considered, VLs (either bilateral or administrated by the MPP) appeared to be applicable to the largest volumes (78%) of ARVs generic procurements in 2013–2014, compared with LCD waivers (21.7%), CLs (0.25%), patent non-assert policies (0.37%) and other non-specified mechanisms (21%). - Patents were less common in lowest income importing countries (about 20% coverage), yet the existence of patents in an exporting country may have large influence upon procurement in those countries. - Overall, LMICs were able to procure generic versions of patented ARV even when they were patented in both the exporter and the importer countries (unlike, for example, in the US where generics for the same medicines were not available at the same time frame).
**Capacity to Pay Analysis**	Assefa et al., 2017 [57]	- The current prices of DAAs (both from generic manufacturers at US$684 and originator firm at US$1200 FOR 12-weeks of treatment) are much more than the median annual income per capita and the annual health budget of most of the seven African LMICs included in the analysis. - To bear the cost of achieving universal coverage for HCV, governments would be required additional health expenditure ranging from a 4% increase to present rates in South Africa to about 400% in Cameroon.

## Data Availability

Not applicable.

## Supplementary Materials

The following supporting information can be downloaded at: https://www.mdpi.com/article/10.3390/pharmaceutics14010048/s1. Table S1: Search terms and strategy. Table S2: List of excluded full-text articles and reasons for exclusions.
