# Supplementary material for: TRIPS to Where? A Narrative Review of the Empirical Literature on Intellectual Property Licensing Models to Promote Global Diffusion of Essential Medicines
Source: Pharmaceutics. 2021 Dec 27;14(1):48. doi: 10.3390/pharmaceutics14010048 (PMC8779122; doi:10.3390/pharmaceutics14010048)
Supplement: Supplementary file 1 [file pharmaceutics-14-00048-s001.zip › pharmaceutics-1513503-SI.pdf]

pharmaceutics

Supplementary Materials: TRIPS to Where? A Narrative Review of the Empirical Literature on Intellectual Property Licensing Models to Promote Global Diffusion of Essential Medicines

Shiri Mermelstein and Hilde Stevens

The structured literature search was conducted using MeSH terms and free text in combination with the search strategy as follows:

Table S1. Targeted search terms and strategy.

Category (AND)	MeSH Terms / Key Words (OR)
Intervention	Intellectual Property (MeSH) licensing<br>Voluntary licensing<br>Collaborative licensing<br>Patent pool
Outcome	Health services accessibility (MeSH)<br>Drug costs (MeSH)<br>Drug prices<br>Generic drugs (MeSH)<br>Drug diffusion<br>Drug uptake<br>Drug affordability

Wildcard (*) was used to maximize search results in databases.

PubMed: (intellectual property licen*[TIAB] OR voluntary licen* [TIAB] OR collaborative licen* [TIAB] OR patent pool* [TIAB]) AND (medicin* OR pharmaceutical* OR drug*) AND (health services accessibility [MeSH] OR costs [MESH] OR pric* OR generic [MESH] OR diffusion [TIAB] OR uptake [TIAB] OR affordability [TIAB])

Scopus: (ALL ("intellectual property licencing" OR "voluntary licencing" OR "collaborative licensing" OR "patent pool") AND ALL (medicin* OR pharmaceutical* OR drug*) AND ALL (health AND services AND accessibility OR costs OR pric* OR generic OR diffusion OR uptake OR affordability))

EconLit: intellectual property licen* OR voluntary licen* OR collaborative licen* OR patent pool* AND drug* or pharmaceutical* or medicin* AND accessibility or access OR costs OR pric* OR generic OR diffusion OR uptake OR affordability

Table S2. List of excluded full-text articles (*n*=24) and reasons for exclusion.

	**I. No Association of Interest: Does not Link VLs and Access to Medicines (*n*=7)**
1	Motari, M.; Nikiema, J.B.; Kasilo, O.M.J.; Kniazkov, S.; Loua, A.; Sougou, A.; Tumusiime, P. The Role of Intellectual Property Rights on Access to Medicines in the WHO African Region: 25 Years after the TRIPS Agreement. *BMC Public Health* **2021**, *21*, doi:10.1186/s12889-021-10374-y.
2	Schröeder, S.E.; Pedrana, A.; Scott, N.; Wilson, D.; Kuschel, C.; Aufegger, L.; Atun, R.; Baptista-Leite, R.; Butsashvili, M.; El-Sayed, M.; et al. Innovative Strategies for the Elimination of Viral Hepatitis at a National Level: A Country Case Series. *Liver International* **2019**, *39*.
3	Tripathy, S. Bio-Patent Pooling and Policy on Health Innovation for Access to Medicines and Health Technologies That Treat HIV/AIDS: A Need for Meeting of [Open] Minds. In *Global Governance of Intellectual Property in the 21st Century: Reflecting Policy Through Change*; **2016**.

4	Iyengar, S.; Tay-Teo, K.; Vogler, S.; Beyer, P.; Wiktor, S.; de Joncheere, K.; Hill, S. Prices, Costs, and Affordability of New Medicines for Hepatitis C in 30 Countries: An Economic Analysis. *PLoS Medicine* **2016**, *13*, doi:10.1371/journal.pmed.1002032.
5	Craddock, S. *Compound Solutions: Pharmaceutical Alternatives for Global Health*; 2017.
6	Zeng, J.; Zhang, W.; Tang, Q. Analysis of the Factors Influencing Enterprise and Government Participation in the Medicines Patent Pool Based on System Dynamics Model. *Iranian Journal of Public Health* **2018**, *47*.
7	Sim, J.; Hill, A. Is Pricing of Dolutegravir Equitable? A Comparative Analysis of Price and Country Income Level in 52 Countries. *Journal of Virus Eradication* **2018**, *4*, doi:10.1016/s2055-6640(20)30311-3.
	**II. No Exposure of Interest: Does not Measure Voluntary Licensing** (*n*=4)
8	Son, K.B.; Kim, C. yup; Lee, T.J. Understanding of for Whom, under What Conditions and How the Compulsory Licensing of Pharmaceuticals Works in Brazil and Thailand: A Realist Synthesis. *Global Public Health* **2019**, *14*, doi:10.1080/17441692.2018.1471613.
9	Ooms, G.; Hanefeld, J. Threat of Compulsory Licences Could Increase Access to Essential Medicines. *The BMJ* **2019**, *365*, doi:10.1136/bmj.l2098.
10	Meiners, C.; Sagaon-Teyssier, L.; Hasenclever, L.; Moatti, J.P. Modeling HIV/AIDS Drug Price Determinants in Brazil: Is Generic Competition a Myth? *PLoS ONE* **2011**, *6*, doi:10.1371/journal.pone.0023478.
11	Flynn, S.; Hollis, A.; Palmedo, M. An Economic Justification for Open Access to Essential Medicine Patents in Developing Countries. *Journal of Law, Medicine and Ethics* **2009**, *37*, doi:10.1111/j.1748-720X.2009.00365.x.
	**III. No Outcome of Interest: Does not Measure Access to Medicines** (*n*=1)
12	Rocha, M. de M.; de Andrade, E.P.; Alves, E.R.; Cândido, J.C.; Borio, M. de M. Access to Innovative Medicines by Pharma Companies: Sustainable Initiatives for Global Health or Useful Advertisement? *Global Public Health* **2020**, *15*, doi:10.1080/17441692.2020.1729391.
	**IV. No Empirical Analysis** (*n*=10)
13	Pandey, E.; Paul, S.B. Affordability versus Innovation: Is Compulsory Licensing the Solution? *International Journal of Risk and Safety in Medicine* **2019**, *30*, doi:10.3233/JRS-195007.
14	Adekola, T.A. Public Health–Oriented Intellectual Property and Trade Policies in Africa and the Regional Mechanism under Trade-Related Aspects of Intellectual Property Rights Amendment. *Public Health* **2019**, *173*, doi:10.1016/j.puhe.2019.04.019.
15	Douglass, C.H.; Pedrana, A.; Lazarus, J. v.; Hoen'T, E.F.M.; Hammad, R.; Leite, R.B.; Hill, A.; Hellard, M. Pathways to Ensure Universal and Affordable Access to Hepatitis C Treatment. *BMC Medicine* **2018**, *16*.
16	Grillon, C.; Krishtel, P.R.; Mellouk, O.; Basenko, A.; Freeman, J.; Mendão, L.; Andrieux-Meyer, I.; Morin, S. Treatment Advocate Tactics to Expand Access to Antiviral Therapy for HIV and Viral Hepatitis C in Low- to High-Income Settings: Making Sure No One Is Left Behind. *Journal of the International AIDS Society* **2018**, *21*, doi:10.1002/jia2.25060.
17	Urias, E. The Contribution of the Pharmaceutical Industry to the Health Status of the Developing World. In *International Business and Management*; **2017**; Vol. 33.
18	Raju, K.D. Compulsory v Voluntary Licensing: A Legitimate Way to Enhance Access to Essential Medicines in Developing Countries. *Journal of Intellectual Property Rights* **2017**, *22*.
19	Bermudez, J.; Hoen, E. 't The UNITAID Patent Pool Initiative: Bringing Patents Together for the Common Good. *The Open AIDS Journal* **2010**, *4*, doi:10.2174/1874613601004020037.
20	Satyanarayana, K.; Srivastava, S. Patent Pooling for Promoting Access to Antiretroviral Drugs (ARVs) – A Strategic Option for India. *The Open AIDS Journal* **2010**, *4*, doi:10.2174/1874613601004020041.
21	Dionisio, D. Medicines Patent Pool: Making the Difference on Access. *Future Virology* **2011**, *6*.
22	Hsu, L. *Trade, Investment, Innovation and Their Impact on Access to Medicines: An Asian Perspective*; **2016**.
	**V. Full Text [book or ebook] Could not be Accessed** (*n*=2)
23	Halliburton, M. *India and the Patent Wars: Pharmaceuticals in the New Intellectual Property Regime*; **2017**.
24	Owoeye, O. *Intellectual Property and Access to Medicines in Africa: A Regional Framework for Access*; **2019**.
